# The Ocular Signs of Migraine
1A Paper read at a Meeting of the Bristol Medico-Chirurgical Society, held at the University of Bristol on January 13th, 1926.


**Published:** 1926

**Authors:** E. R. Chambers

**Affiliations:** Ophthalmic Surgeon, Bristol Royal Infirmary


### THE OCULAR SIGNS OF MIGRAINE.[1]

BY

E. R. Chambers, F.R.C.S.,

*Ophthalmic Surgeon, Bristol Royal Infirmary.*

---

Visual phenomena form one of the most constant and striking features in migraine attacks. Gowers points out that the prodromata of migraine are a functional disturbance in the sensory organ, and so is the headache which succeeds. By the headache is implied a coarse disturbance consisting of pain, often intense, or, on the other hand, slight and of short duration, whilst in some cases the prodromata occur alone, not being followed by headache. These premonitory symptoms, although sensory, do not include pain. The sense of sight is very often involved, sometimes by loss, partial or general, sometimes by an elaborate display of its higher crude functions, in light, colour or apparent motion.

Of the processes which induce the premonitions we have only hypothesis to rest on; there is clearly a disturbance of function in certain parts of the cerebral hemisphere, probably the cortex, which is the subsequent seat of the pain. The disturbance in function is apparently crude, and seems as it were to ripple through the centre, leaving an inhibited condition, which quickly passes away. It is possible that cerebral anæmia due to vaso-motor spasm is the cause of

[1] A Paper read at a Meeting of the Bristol Medico-Chirurgical Society, held at the University of Bristol on January 13th, 1926.

these prodromal symptoms, and in support of this is the fact that the retinal arteries are markedly contracted during the prodromal stage.

Many of the ocular manifestations of migraine closely resemble the visual aura present in epilepsy, and in rare cases the conditions co-exist. The prodromata of migraine are, however, never followed by any motor symptoms, except for some muscular twitchings in those cases in which the headache is very severe.

An examination of the eyes during the prodromal symptoms or the attack itself reveals little. The pupils are usually dilated but react well to light and accommodation. In some cases the pupils may be in a state of hippus, *i.e.* they may be seen with the naked eye to contract and dilate rhythmically quite apart from any change in the intensity of the light.

The only constant manifestation of migraine seen on an examination of the fundus is a definite contraction of the arteries; and this condition is particularly noticeable if one follows the retinal vessels towards the periphery. It is most marked during the prodromal stages, and is less during the actual attack. The retinal veins are unaffected, or, if anything, dilated. In the cases I have examined I have not seen one case in which the vessel walls showed anything of the nature of arterio-sclerosis, and, if we assume that the retinal vessels are any guide to the condition of other vessels from the same source which go to supply the brain, it would point to these arteries being healthy, but a similar contraction may be present in the cerebral vessels, as is so clearly seen in those which supply the retina.

By far the commonest premonitory symptom of migraine is the appearance in the vision of spots; these vary in number, are usually small and have an irregular outline. Coloured spots are not uncommon, nor are those whose centre is black and the edges either coloured or glittering. Frequently, usually just before the headache commences, there appears amongst these spots a comparatively large and glistening ball, situated at or near the centre of vision. Such was the condition in a case I recently saw of a young man who for some hours before the headache commenced could, with difficulty, carry on his work as a clerk, owing to the fact that, as he put it, he could not see through the myriads of spots when looking at white paper, and that for another hour or so before the attack he was entirely prevented from reading or writing owing to the large glistening ball which occluded his central vision. I was able to map out the visual fields in this case, and found a small central scotoma for white and colours, whilst a much larger area of the field was also blind for red and green.

Sometimes in place of the spots in the vision the patient complains that objects appear as if they were seen through a haze, such as rises from the earth on a hot day. In these cases I have found no abnormality in the visual fields.

The so-called scintillating scotoma is another and very characteristic ocular manifestation of migraine; it may precede the attack by a day or only by a few hours. The duration of these attacks is usually short, not lasting more than an hour or so, only to be followed by a typical attack of migraine; but in exceptional cases the blindness produced by the scotoma may be of very much longer duration. Thus in a case I saw the other day a young woman had been shopping in town, when suddenly and without any warning flashes of light appeared before her eyes, rendering her in the course of a few minutes quite blind. When I saw her both eyes were affected, and the vision amounted to not more than counting fingers at two feet. The fundus on examination was normal, except for a contraction of the retinal arteries. There was some vertigo but no true headache, the pupils were dilated but reacted well to light. She gave a typical history of migraine, and said that it had been in her family for generations. She had, in past attacks, had flashes of light and diminution of vision before the headache commenced, but she had never experienced anything like the present condition. I watched her for three hours; after the first hour the headache commenced and vision began to clear, and by taking a rough visual field I found that the scotoma was gradually clearing from the periphery to the centre. At the end of the third hour both vision and visual fields were normal, but by now she had an intense headache with vomiting.

The foregoing case at first suggested one of the fairly common functional varieties of blindness, but the past history and the subsequent vomiting is, I think, sufficient evidence that the condition was due to migraine.

Other cases are met with in which coloured zig-zags appear on one side of the visual field, with at first a reduction of vision on this side only. Slowly the spectrum passes to the opposite side of the field, until vision is completely lost. Or the coloured zig-zags may be intermittently scattered over the visual fields, and in these cases vision is lost and regained alternately for a while until either temporary blindness results or the prodromata cease and the headache commences.

The above types of scotomata are undoubtedly of central origin, as they always affect both eyes with normal fundi. It is probable that severe symptoms of such short duration could only be caused by disturbances of the circulation, the transient character indicating some disorder of the innervation of the vessels, and not disease. The circulatory disturbance sets up an irritation of the optical elements in the occipital lobe, and this irritation, by the natural laws of projection, is referred to the external world and appears in the form of a scintillating scotoma.

Perhaps the most interesting of all the ocular manifestions of migraine is in those cases in which the prodromata are in the form of a transient hemianopia. These are usually of the homonymous or lateral type, so that the patient is unable to see objects to the right or the left. More rarely the upper or lower halves of the fields are involved, the so-called altitudinal hemianopsia. This type of partial blindness is usually rapid in onset, and except for some slight dizziness the patient is usually quite well; its duration is usually short, to be followed by the typical headache. Cases are, however, recorded in which the hemianopsia appeared with the headache and even persisted after it. The visual fields in these cases rarely show a complete hemianopsia, *i.e.* the dividing-line between the blind and seeing halves of the fields does not, as a rule, pass through the fixation point. Sometimes in the blind halves of the field there may be flashes of light or sensation of colours, or the edges of the scotoma may be zig-zagged and glittering.

Patients who have had migraine for years with visual prodromata, but who have never had the homonymous type of scotoma, may suddenly be attacked in this way, or the homonymous type may be the usual phenomenon with them.

Thus a middle-aged lady, who stated that she had suffered from sick-headaches since she was a girl, suddenly experienced flashes of light in her eyes; this was followed by zig-zag lines with coloured edges. She happened to look at the face of a clock, and found that she could not see anything on her right side. She was well accustomed to the flashes of light as prodromata of previous attacks, but she had never seen the zig-zag lines with coloured edges, nor had she ever experienced failure of half her vision.

A visual field in this case showed a marked right homonymous hemianopsia, not involving the fixation point. Her subsequent history was a very severe attack of headache and vomiting, but when I saw her some days after the attack there was no sign of any contraction of the visual fields, the acuity of vision being normal.

Gowers and Ormond quote cases in which the hemianopsias of migraine have not been recovered from, and have persisted for years, if not permanently. Gordon Holmes also mentions a similar case in a woman, aged 30, married and healthy. Wassermann negative. No heart trouble nor Bright's disease. The pulse tension was good, but she had suffered from childhood with severe attacks of characteristic ophthalmic migraine, unilateral headache with typical subjective visual phenomena. After one of these severe attacks she was lying down in consequence of the headache, and when she got up she found that the blindness, which she said generally preceded the onset of the headache, persisted for a much longer time than usual. She was found to have complete left homonymous hemianopsia. He had watched the case for three or four years, but the condition still persisted. He had not been able to account for it. These cases seem to suggest that simple migraine may be followed by permanent lesions involving the cortical area. If migraine is due to vasomotor spasm causing anæmia in the region supplied by those vessels, and the subsequent dilatation of the arteries causes the pain, it may be that the temporary spasm has been sufficient to cause a permanent thrombosis in the vessels in question. The retinal arteries can be seen, and are, as I have pointed out, always markedly contracted during the prodromal symptoms of migraine.

Finally, we come to a group of cases which are rarely met with, and are characterised, not by a scotoma, but by paralysis of a recurrent type affecting the muscles supplied by the third nerves. Charcot labelled these cases "Ophthalmoplegic Migraine," owing to the fact that muscle paralyses are always associated with symptoms resembling migraine. These paralyses are at first temporary, passing away as the attack of migraine subsides, but later they tend to become permanent.

In most cases migraine, without paralysis of the ocular muscles, has existed for years, then, during an attack of unusual severity, the ocular palsy appears and thereafter occurs in subsequent attacks. It remains limited to the side in which it first occurred, and is seldom, if ever, bilateral. The condition affects the sexes equally, and usually begins in early life. The paralysis outlasts the attack of migraine, and may take from one to seven days to recover. The extra-ocular muscles supplied by the third nerve are those usually involved, resulting in an external squint with some ptosis, whilst the intra-ocular muscles usually escape.

It is perhaps unfortunate that these cases should have been classified by Charcot as ocular symptoms of migraine, as his classification is followed to the present day; for although the headache and vomiting are so suggestive of migraine, careful inquiry fails to elicit any of the prodromal ocular symptoms, such as scotomata, hemianopsia, or visual spectra. It is probable that these palsies are not due to migraine, but are organic in origin.

As regards the treatment of migraine, I am, fortunately, only concerned with that of the eyes. This resolves itself into correcting any errors of refraction and dealing with abnormalities in the muscle balance of the eyes.

As regards errors of refraction, I am quite convinced that migraine cannot be cured by the most carefully prescribed glasses, but there is also no doubt that the interval can be prolonged between the attacks.

The correction of large errors of refraction seems to do little to help the patient, probably because there is very little strain in these cases, as the patient finds it so difficult to see clearly that he gives up trying, but it is to the small errors of refraction, particularly astigmatism, that we must so carefully draw our attention if we are to do our best for the patient. It is not the slightest use testing these patients' vision with a test-card, finding it normal, and so assuming that there is no error of refraction. All cases of migraine should have their pupils dilated and a careful retinoscopy done. Small errors will thus be detected that by any other method would escape notice.

As regards the correction of any abnormality in muscle balance, this is probably more important than the correction of errors of refraction. The strain produced is greater, and the result from wearing even a very small prism is such that the possibility of an error of this nature should never be overlooked.

### DISCUSSION.

COL. LISTER said that in his experience of migraine the order of the symptoms was sometimes reversed; that is, the typical ball of light was noted in the attacks of early youth, but disappeared in later life. He mentioned a case.

Dr. Charles asked why Mr. Chambers considered the cerebral cortex was involved in migraine. For himself he regarded migraine as due to pituitary swelling. We knew that at times alterations in the thyroid or ovary caused changes in the pituitary. The anatomical relations appeared to favour his view. The vascular and nervous structures, including sympathetic supply in the immediate neighbourhood, would explain the local symptoms, glycosuria, polyuria, anarthria, diarrhœa seemed to point to the pituitary. He found that pituitary lesions generally produced not bitemporal hemianopsia but homonymous.

Dr. Carleton agreed with the last speaker that the cortex could not be the site of the lesion, especially if we were to include migraine of the Charcot type, with ophthalmoplegia. The latter cannot be cortical. He suggested that pure perception of light was of the nature of a protopathic sensation; vision as we knew it was epicritic. Spasm of the ophthalmic artery suppressed the latter and released the former to consciousness. However, he could not conceive that a pituitary swelling (apart from a gross enlargement) could affect the structures in, and in the outer wall of, the cavernous sinus. In his experience the hemianopsia of pituitary tumour was bitemporal.

Mr. Chambers replied. He could not accept Dr. Charles's suggestion that pituitary swelling explained the syndrome of migraine. Nor did he think that the Charcot type was a true migraine. He agreed that blindness might quite well be retinal, but this would not explain the cases in which a hemianopsia occurred.

